# Empirically Derived Dehydration Scoring and Decision Tree Models for Children With Diarrhea: Assessment and Internal Validation in a Prospective Cohort Study in Dhaka, Bangladesh

**DOI:** 10.9745/GHSP-D-15-00097

**Published:** 2015-08-18

**Authors:** Adam C Levine, Justin Glavis-Bloom, Payal Modi, Sabiha Nasrin, Soham Rege, Chieh Chu, Christopher H Schmid, Nur H Alam

**Affiliations:** ^a^​ The Warren Alpert Medical School of Brown University, Providence, RI, USA; ^b^​ International Centre for Diarrhoeal Disease Research, Bangladesh (icddr,b), Dhaka, Bangladesh; ^c^​ Brown University School of Public Health, Department of Biostatistics, Providence, RI, USA

## Abstract

**Introduction:**

Diarrhea remains one of the most common and most deadly conditions affecting children worldwide. Accurately assessing dehydration status is critical to determining treatment course, yet no clinical diagnostic models for dehydration have been empirically derived and validated for use in resource-limited settings.

**Methods:**

In the Dehydration: Assessing Kids Accurately (DHAKA) prospective cohort study, a random sample of children under 5 with acute diarrhea was enrolled between February and June 2014 in Bangladesh. Local nurses assessed children for clinical signs of dehydration on arrival, and then serial weights were obtained as subjects were rehydrated. For each child, the percent weight change with rehydration was used to classify subjects with severe dehydration (>9% weight change), some dehydration (3–9%), or no dehydration (<3%). Clinical variables were then entered into logistic regression and recursive partitioning models to develop the DHAKA Dehydration Score and DHAKA Dehydration Tree, respectively. Models were assessed for their accuracy using the area under their receiver operating characteristic curve (AUC) and for their reliability through repeat clinical exams. Bootstrapping was used to internally validate the models.

**Results:**

A total of 850 children were enrolled, with 771 included in the final analysis. Of the 771 children included in the analysis, 11% were classified with severe dehydration, 45% with some dehydration, and 44% with no dehydration. Both the DHAKA Dehydration Score and DHAKA Dehydration Tree had significant AUCs of 0.79 (95% CI = 0.74, 0.84) and 0.76 (95% CI = 0.71, 0.80), respectively, for the diagnosis of severe dehydration. Additionally, the DHAKA Dehydration Score and DHAKA Dehydration Tree had significant positive likelihood ratios of 2.0 (95% CI = 1.8, 2.3) and 2.5 (95% CI = 2.1, 2.8), respectively, and significant negative likelihood ratios of 0.23 (95% CI = 0.13, 0.40) and 0.28 (95% CI = 0.18, 0.44), respectively, for the diagnosis of severe dehydration. Both models demonstrated 90% agreement between independent raters and good reproducibility using bootstrapping.

**Conclusion:**

This study is the first to empirically derive and internally validate accurate and reliable clinical diagnostic models for dehydration in a resource-limited setting. After external validation, frontline providers may use these new tools to better manage acute diarrhea in children.

## Abstract (precis)

The DHAKA Dehydration Score and the DHAKA Dehydration Tree are the first empirically derived and internally validated diagnostic models for assessing dehydration in children with acute diarrhea for use by general practice nurses in a resource-limited setting. Frontline providers can use these new tools to better classify and manage dehydration in children.

## INTRODUCTION

Despite major advances in prevention and management, diarrhea remains one of the most common and most deadly conditions affecting children today. Each year, children worldwide experience 1.7 billion diarrheal episodes, leading to 124 million outpatient visits and 9 million hospitalizations.^1^^,^^2^ While most episodes of diarrhea in children resolve uneventfully, approximately 36 million cases each year progress to severe disease, resulting in 700,000 deaths, or 10% of all child deaths worldwide.^3^

As the severity of diarrhea in children varies widely, accurately assessing dehydration status is critical to prevent mortality and morbidity. While children with severe dehydration require immediate intravenous fluids (IVF) to prevent hemodynamic compromise, organ ischemia, and death, children with mild to moderate dehydration have shorter hospital stays and fewer adverse events when treated with oral rehydration solution (ORS) alone.^4^ Accurately assessing dehydration status can also improve the cost-effectiveness of diarrhea treatment in resource-limited settings by limiting the use of expensive and resource-intensive IVF.

 Global health authorities therefore recommend classifying children with acute diarrhea into 3 categories based on their initial clinical presentation: no dehydration, some dehydration, or severe dehydration.^5^^-^^8^ Children with no dehydration should receive only expectant management, those with some dehydration should be rehydrated using ORS, and those with severe dehydration should be resuscitated with IVF.

Treatment of children with acute diarrhea varies depending on their dehydration status.

Unfortunately, the diagnostic tools available to clinicians in resource-limited settings to assess the degree of dehydration in children with diarrhea are limited. A large meta-analysis found that no individual clinical sign, symptom, or laboratory test demonstrated adequate sensitivity, specificity, and reliability for detecting dehydration in children.^9^ The World Health Organization (WHO) Integrated Management of Childhood Illness (IMCI) guidelines recommend using a combination of clinical signs to classify children as having no, some, or severe dehydration (Supplementary Appendix 1).^10^ However, the WHO algorithm was developed based largely on expert opinion, and recent studies have not found it to be an accurate predictor of dehydration in children.^11^^-^^13^

Clinicians in resource-limited settings have limited diagnostic tools to assess dehydration severity in children with diarrhea.

To date, no clinical diagnostic model for dehydration in children with diarrhea has been empirically derived and validated for use in a low-income country. This study aims to derive such a clinical diagnostic model for dehydration in children, which can be used by nurses and other non-physician health workers to determine the best management strategy for children with acute diarrhea worldwide.

## METHODS

### Study Design

Data were collected as part of the Dehydration: Assessing Kids Accurately (DHAKA) study, a prospective cohort study of children presenting to the International Centre for Diarrhoeal Disease Research, Bangladesh (icddr,b). The DHAKA study was preregistered at ClinicalTrials.gov (NCT02007733), and ethical approval was obtained from the Lifespan Institutional Review Board and the icddr,b Ethical Review Committee.

### Study Setting

Enrollment for this study took place in the icddr,b rehydration (short stay) unit between February and June 2014. With a catchment area of more than 17 million people, icddr,b provides free clinical services to the population of Dhaka and surrounding rural and suburban districts.^14^ Approximately 90% of children present primarily to the icddr,b rehydration ward, with the remainder referred there from other facilities.

### Study Population

All children under 60 months of age presenting with acute diarrhea were eligible for enrollment. Study staff randomly selected children for screening on arrival 24 hours per day, 7 days per week by pulling blue (selected) or white (not selected) marbles from a blind pouch. Once selected, study staff determined if the child met any of the predefined exclusion criteria:

- Fewer than 3 loose stools per day
- Diarrhea lasting longer than 14 days
- A diagnosis other than gastroenteritis as determined by the treating physician
- Prior enrollment in the study

For patients who did not meet exclusion criteria, research staff approached the parent/guardian, explained the risks and benefits of the study, and obtained consent in the local language, Bengali.

### Choosing Diagnostic Variables

Candidate diagnostic variables, which included signs and symptoms typically associated with dehydration in children, were chosen *a priori* based on their prior performance in published studies, including their accuracy and reliability, and in consultation with expert clinicians at icddr,b regarding their generalizability to resource-limited settings. Ten potential clinical diagnostic variables were identified in this manner:

- General appearance
- Skin pinch
- Sunken eyes
- Tears
- Radial pulse
- Deep breathing
- Extremity warmth
- Heart rate
- Mucous membranes
- Capillary refill

Ten clinical signs typically associated with dehydration in children were identified and measured in this study.

Each of these 10 variables was categorized into 3 levels of severity. In consultation with expert clinicians at icddr,b, detailed procedures were developed to ensure the objective measurement of each clinical sign (Supplementary Appendix 2).

### Staff Training

Local general practice nurses with 4–6 years of clinical experience collected all data for this study. These research nurses were hired outside of the icddr,b clinical nursing pool specifically to collect data for this study and had no other clinical responsibilities during the study period. Prior to the start of the study, they received 5 days of training in all study procedures. This included an in-depth review of the clinical signs of dehydration, with explicit didactic and video instruction in how to appropriately assess each sign as outlined in Supplementary Appendix 2. Nurses also received practical training, with each nurse performing a full assessment of at least 10 children in the rehydration unit at icddr,b under the guidance of the primary investigator prior to the start of the study.

### Data Collection

#### Baseline Data

Immediately after obtaining informed consent, children were undressed and weighed to the nearest tenth of a kilogram using an electronic scale. If a child received intravenous fluid before baseline weight was obtained, study staff recorded the amount of fluid received prior to measurement.

Subjects were then assessed clinically by a study nurse for presence of the 10 clinical signs of dehydration noted above. Subjects were also assessed clinically by a second study nurse when available, blinded to the exam performed by the first nurse.

Study nurses collected baseline historical and demographic data for each child from their parent/guardian including location, age, gender, days of diarrhea, diarrheal episodes in the past 24 hours, and type of diarrhea (bloody, watery, rice water). Study nurses also measured mid-upper arm circumference (MUAC) on arrival to the nearest millimeter using a standard measuring tape.

#### Follow-Up Data

Patients were then treated according to standard icddr,b protocols. Emergent care was not delayed for consent, measurements, or weights. All enrolled children received a unique bar-coded study label on their admission card, matching the bar code on their case report forms, to ensure accurate collection of all data.

Patients were weighed every 8 hours, on the same scale and without clothing, to determine their post-hydration stable weight, which was used as a proxy for their pre-illness weight. Children who did not achieve a stable weight prior to discharge were telephoned daily after discharge until their diarrhea resolved, then asked to return for a post-illness weight check.

### Data Analysis

#### Baseline Data

Baseline historical, demographic, and nutritional data were summarized for all children enrolled in our study. The proportion of children with undernutrition was calculated using a MUAC <115 mm for severe acute malnutrition (SAM) and MUAC 115–125 mm for moderate acute malnutrition (MAM).^15^

#### Analysis of Dehydration Status

For each patient enrolled, we averaged the 2 highest consecutive weight measurements that differed by less than 2% to determine a stable weight, as described in the literature.^16^ In general, children with dehydration rapidly gain weight as they are rehydrated until they achieve their pre-illness weight, or stable weight, at which point they will stop gaining weight as their kidneys diurese excess fluid. For each patient with a valid stable weight, we calculated the percent weight change with rehydration, our criterion standard for percent dehydration, using the following formula:

Percent Dehydration = [(Stable Weight – Admission Weight) / Stable Weight] * 100

Dehydration status was based on the child’s stable weight after rehydration and the weight at admission.

For children who did not achieve a stable weight prior to discharge, their post-illness weight was used instead of their stable weight in the formula above to calculate the percent dehydration. We then calculated the proportion of children with severe dehydration (>9% weight change), some dehydration (3–9%), and no dehydration (<3%). Children who lost significant weight during their stay in the rehydration unit, suggesting inadequate hydration in the face of ongoing diarrhea or an erroneous admission weight, were excluded from analysis, as their dehydration category could not be determined.

#### Analysis of Diagnostic Variables

We calculated the proportion of children presenting with each of the 10 signs of dehydration defined previously. Two signs, slow capillary refill and cool extremities, were found to be present in less than 5% of cases and were therefore excluded from analysis. While rare signs might still be strong diagnostic criteria, they are unlikely to be efficient criteria (since they require effort to collect and are unlikely to influence the patient’s diagnosis in the vast majority of cases). Additionally, it would be difficult for less experienced practitioners to reliably identify clinical signs encountered so infrequently.

For the remaining 8 clinical signs, we performed a bivariate analysis to assess the association of each variable with the true dehydration status of the child based on our criterion standard. We also assessed the test characteristics of each diagnostic variable for the presence of severe dehydration in children, based on our criterion standard. Finally, for the subset of children with a repeat exam, we calculated the inter-rater reliability of each diagnostic variable using Cohen’s Kappa (weighted).

#### Derivation of Clinical Diagnostic Models

Standard guidelines from the literature, including the recently published “Transparent Reporting of a multivariable prediction model for Individual Prognosis Or Diagnosis” (TRIPOD) guidelines, were used to develop clinical diagnostic models for dehydration severity.^17^^-^^20^ Primary analyses were performed using R 3.0.2 (R Development Core Team, Vienna, Austria). Figures were produced using STATA 12.0 (STATA Corp, College Station, TX, USA).

The 8 candidate diagnostic variables were entered into an ordinal logistic regression (proportional odds) model for the outcome of dehydration category (none, some, or severe). A stepwise backward selection algorithm was used to derive a final clinical diagnostic model using a stopping rule of *P* < .10. In this way, clinical variables that were only weakly associated with the true dehydration status of the child, after controlling for all other clinical variables, were sequentially dropped until only statistically significant variables remained. An alternative stepwise selection algorithm using the Akaike Information Criterion (AIC) was also performed, which is a different means of selecting variables that does not rely on the *P* value alone to select the final variables for the model. Since only 3 patients were missing any data on clinical diagnostic variables (1 patient was missing data on heart rate and 2 patients were missing data on tears), we used case-wise deletion to handle missing data instead of single or multiple imputation. The final logistic regression model was then converted into a scoring system by ordering the final clinical variables in tabular format and converting the log odds ratio for each variable into an integer score as described previously in the literature.^19^ We refer to this new scoring system as the Dehydration: Assessing Kids Accurately (DHAKA) Dehydration Score.

Logistic regression modeling was used to develop a dehydration scoring system based on clinical signs, called the DHAKA Dehydration Score.

We developed a second clinical diagnostic model using recursive partitioning, an alternative method that may perform better than standard regression analysis when all components of the model can be broken down into a series of yes/no questions and when there are important interactions among the predictors.^18^^,^^21^ Using the R *tree* package, we performed recursive binary splitting on our dataset using our 8 candidate variables to grow an initial decision tree. We then performed cost complexity pruning to determine the cost complexity factor (α) for trees of different sizes. Finally, we performed 10-fold cross validation to determine the level of α that minimized the average mean squared prediction error in our cross validation test sets to select the sub-tree that was least likely to overfit the data and most likely to perform similarly in a new dataset. We refer to this new clinical decision tree as the Dehydration: Assessing Kids Accurately (DHAKA) Dehydration Tree.

Recursive partitioning was used to develop a dehydration decision tree based on clinical signs, called the DHAKA Dehydration Tree.

#### Model Assessment

We assessed the discrimination of both our DHAKA Dehydration Score, derived using ordinal logistic regression, and our DHAKA Dehydration Tree, derived using recursive partitioning, by calculating the area under the receiver operating characteristic (ROC) curves (AUC), or c-statistic, for each model against the true dehydration category of the child. The shape of a ROC curve and the AUC help estimate the discriminative power of a diagnostic test. The closer the curve is located to the upper-left hand corner of a graph and the larger the area under the curve, the better the test is at discriminating between people with the disease (in this case, dehydration) and without the disease. The AUC can have a value between 0 and 1; a perfect diagnostic test has an AUC of 1.0 while a non-discriminating test has an area of 0.5.

The test characteristics for the DHAKA Dehydration Score at its best cut-points and the DHAKA Dehydration Tree were also assessed against the true dehydration category of the child. We also assessed the inter-rater reliability of each model by testing its agreement between the initial exam and repeat exam for the subset of children that had repeat exams using Cohen’s Kappa (weighted).

#### Model Validation

In the absence of an external validation cohort, internal validation was performed using the bootstrap method to assess the optimism of our clinical diagnostic models.^17^^,^^18^ In this context, optimism refers to how much better a diagnostic model performs in the population in which it was derived compared with a new population in which it is validated. A bootstrap (with replacement) sample was randomly selected from our study population and used to derive both models again using the same algorithms used to derive the original models. The AUCs for these models were calculated both in the bootstrap sample and in the full dataset. This process was repeated 1,000 times, and the average differences between the AUCs for the bootstrap samples and the full dataset were used to calculate an unbiased estimate of the optimism of the original AUCs calculated for both models. The optimism represents the amount by which the AUC in our study population would be expected to exceed the AUC in a new test population. A low optimism score suggests the model would perform as well in a new population as in the current study population.

Internal validation was performed to assess how well the new clinical diagnostic models would perform in a new population other than the one in which they were derived.

#### Sample Size

While there is no formal method for calculating the study sample size for the development of a clinical diagnostic model, a general rule of thumb in the literature calls for at least 10 positive events per variable (EPV) considered for the model, although more recent statistical research has suggested that 5 EPV may be sufficient.^17^^,^^18^^,^^22^ Given 8 candidate variables, each with 2 levels of comparison, this would require a minimum of 80 positive outcomes, or 80 children with severe dehydration, to achieve 5 EPV.

## RESULTS

### Enrollment and Baseline Characteristics

**FIGURE 1:**
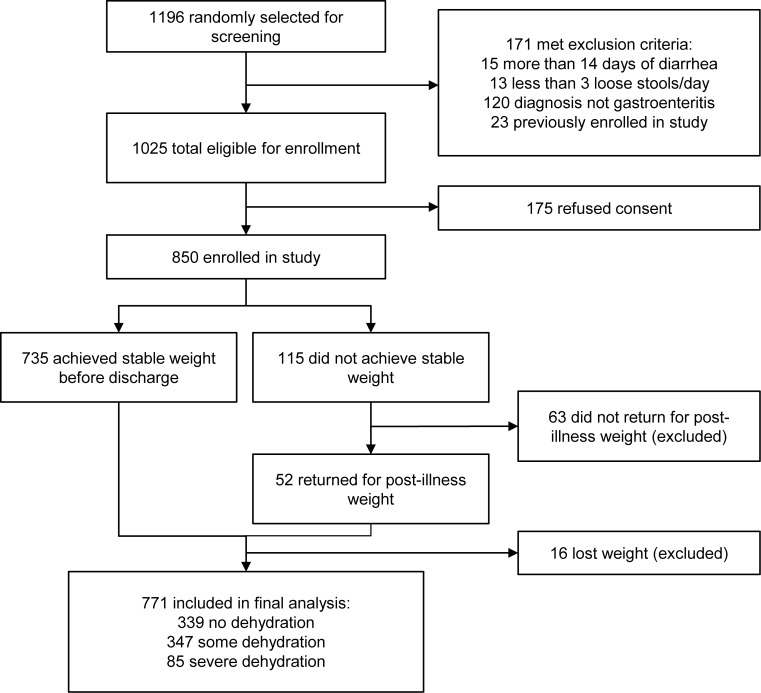
Flowchart for DHAKA Study Enrollment

Of the 1,025 eligible patients randomly selected, 850 were enrolled in the study and 771 were included in the final analysis (Figure 1). Among the 850 enrolled subjects, there were no significant differences in baseline demographic, historical, or anthropometric characteristics between those included and excluded from analysis (Table 1).

**TABLE 1:** Baseline Characteristics of Enrolled Children <60 Months of Age With Acute Diarrhea, Dhaka, Bangladesh, 2014 (N = 850)

	Included in Final Analysis (n = 771)	Excluded, Lost Weight (n = 16)	Excluded, No Final Weight (n = 63)	*P* value
Age in months, median (IQR)	15 (9–29)	18 (13–29)	22 (12–36)	.07^a^
Sex				.84^b^
Female, No. (%)	336 (44)	6 (38)	26 (41)	
Male, No. (%)	435 (56)	10 (62)	37 (59)	
Home district				.99^b^
Urban (Dhaka), No. (%)	478 (62)	14 (88)	45 (71)	
Rural/suburban, No. (%)	293 (38)	2 (12)	18 (29)	
Nutritional status (MUAC)				.30^b^
No acute malnutrition, No. (%)	614 (80)	16 (100)	53 (84)	
Moderate acute malnutrition (MAM), No. (%)	121 (16)	0 (0)	7 (11)	
Severe acute malnutrition (SAM), No. (%)	35 (4)	0 (0)	3 (5)	
Days of diarrhea prior to arrival, median (IQR)	2 (1–4)	2 (1.5–3.5)	2 (1–3)	.13^a^
Loose stools in past 24 hours, median (IQR)	15 (10–20)	15 (11–20)	15 (10–20)	.79^a^
Diarrhea type				.69^b^
Watery, No. (%)	448 (58)	12 (75)	36 (57)	
Rice-water, No. (%)	317 (41)	4 (25)	27 (43)	
Bloody, No. (%)	4 (1)	0 (0)	0 (0)	

Abbreviation: IQR, interquartile range; MUAC, mid-upper arm circumference.

^a^​ Equality of medians.

^b^​ Chi-square test.

### Dehydration Status

The median percent weight change with rehydration was 4% (interquartile range [IQR] = 1%, 7%). Of the 771 children included in the final analysis, 85 (11%) children were classified with severe dehydration, 347 (45%) with some dehydration, and 339 (44%) with no dehydration. Median time from arrival to stable weight was 14 hours (IQR = 11, 19; n = 735), and median time from arrival to post-illness weight was 87 hours (IQR = 56, 99; n = 52). About one-quarter (28%) of children received fluids prior to their initial weight, with the median amount of fluid received just 1.5 ml/kg (IQR = 1.0, 2.7).

### Association of Clinical Signs With Severe Dehydration

The median time from subject arrival to assessment of clinical signs was 4 minutes (IQR = 2, 5). Approximately half of study subjects (n = 419) had a repeat clinical exam performed, with a median time of 6 minutes (IQR = 5, 8) between exams. All 8 clinical variables were significantly associated with the presence of severe dehydration in bivariate analysis, although their individual accuracy and reliability varied (Table 2). For example, sunken eyes had a sensitivity of 94% but a specificity of only 13%, resulting in a positive predictive value (PPV) of just 12% but a negative predictive value (NPV) of 95%.

All 8 clinical variables assessed were significantly associated with severe dehydration status in bivariate analysis.

**TABLE 2:** Association of Clinical Signs With Severe Dehydration in Bivariate Analysis

	Sensitivity	Specificity	PPV	NPV	LR+	LR−	Reliability	Chi-Square	*P* Value
Eyes							0.60	61.54	<.001
Sunken	0.94	0.13	0.12	0.95	1.08	0.46			
Very sunken	0.47	0.87	0.31	0.93	3.55	0.61			
General appearance							0.72	64.01	<.001
Restless/irritable	0.84	0.55	0.19	0.96	1.84	0.30			
Lethargic/unconscious	0.62	0.77	0.25	0.94	2.69	0.49			
Heart rate							0.47	10.28	.006
Fast	0.59	0.57	0.15	0.92	1.38	0.72			
Very fast	0.02	0.99	0.33	0.89	4.04	0.98			
Mucous membranes							0.42	18.79	<.001
Dry/sticky	0.88	0.34	0.14	0.96	1.35	0.34			
Very dry	0.02	0.99	0.25	0.89	2.69	0.99			
Radial pulse							0.60	40.77	<.001
Decreased	0.64	0.71	0.21	0.94	2.17	0.52			
Weak	0.38	0.84	0.23	0.92	2.37	0.74			
Respirations							0.58	35.08	<.001
Deep	0.61	0.69	0.20	0.94	2.00	0.56			
Very deep	0.07	0.98	0.33	0.90	4.04	0.95			
Skin pinch							0.71	69.18	<.001
Slow	0.85	0.53	0.18	0.97	1.79	0.29			
Very slow	0.31	0.93	0.35	0.92	4.37	0.75			
Tears							0.63	54.28	<.001
Decreased	0.85	0.52	0.18	0.96	1.75	0.30			
Absent	0.29	0.92	0.30	0.91	3.43	0.78			

Abbreviations: LR−, negative likelihood ratio; LR+, positive likelihood ratio; NPV, negative predictive value; PPV, positive predictive value.

### Logistic Regression to Derive the DHAKA Dehydration Score

Stepwise backward selection of our full ordinal regression model produced a final model with 4 variables:

- General appearance
- Skin pinch
- Tears
- Respirations

The DHAKA Dehydration Score comprised 4 clinical signs: general appearance, skin pinch, tears, and respiration.

The regression coefficients for each level of each of these variables were converted into integer scores, producing a 12-point scoring system (Table 3). Alternative selection using the AIC instead of a *P*-value rule produced an identical final scoring system.

**TABLE 3:** 12-Point DHAKA Dehydration Scoring System With Assigned Dehydration Categories

Clinical Sign	Finding	Points
General appearance	Normal	0
	Restless/irritable	2
	Lethargic/unconscious	4
Respirations	Normal	0
	Deep	2
Skin pinch	Normal	0
	Slow	2
	Very slow	4
Tears	Normal	0
	Decreased	1
	Absent	2
**DHAKA Dehydration Score Categories**		**Points**
No dehydration		0–1
Some dehydration		2–3
Severe dehydration		≥4

**FIGURE 2:**
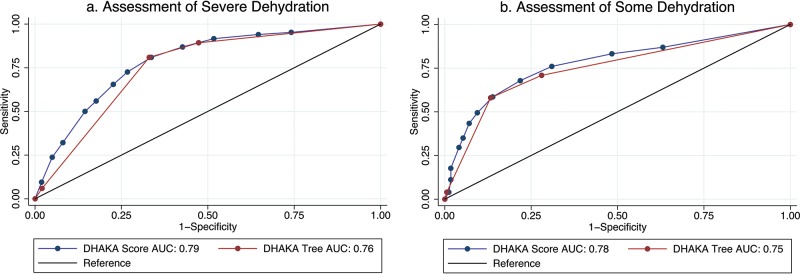
Receiver Operating Characteristic (ROC) Curves of the DHAKA Dehydration Score and DHAKA Dehydration Tree Abbreviation: AUC, area under the receiver operating characteristic curve.

The AUC for this new DHAKA Dehydration Score was 0.79 (95% CI = 0.74, 0.84) for severe dehydration and 0.78 (95% CI = 0.74, 0.81) for some (any) dehydration (Figure 2). For those children with a repeat clinical exam, the DHAKA Dehydration Score had 90% agreement between independent raters, with a Cohen’s Kappa of 0.75 (95% CI = 0.66, 0.85). Table 4 demonstrates the proportion of children with no, some, or severe dehydration by DHAKA Dehydration Score category, and Table 5 demonstrates the test characteristics of the DHAKA Dehydration Score for assessing some and severe dehydration in children. For example, the DHAKA Dehydration Score had a sensitivity of 87% and a specificity of 57%, a positive likelihood ratio (LR+) of 2.0, and a negative likelihood ratio (LR-) of 0.23 for the outcome of severe dehydration.

The DHAKA Dehydration Score was significantly accurate in diagnosing severe and any dehydration.

**TABLE 4:** Assigned DHAKA Dehydration Score and DHAKA Dehydration Tree Categories Compared With True Dehydration Status, No. (%)

	DHAKA Dehydration Score Category			DHAKA Dehydration Tree Category		
True Dehydration Status	No Dehydration (n = 247)	Some Dehydration (n = 156)	Severe Dehydration (n = 365)	No Dehydration (n = 369)	Some Dehydration (n = 106)	Severe Dehydration (n = 296)
No dehydration	175 (71)	91 (58)	73 (20)	244 (66)	50 (47)	45 (15)
Some dehydration	67 (27)	59 (38)	219 (60)	116 (31)	49 (46)	182 (61)
Severe dehydration	5 (2)	6 (4)	73 (20)	9 (2)	7 (7)	69 (23)

**TABLE 5:** Test Characteristics for DHAKA Dehydration Score and DHAKA Dehydration Tree

Clinical Diagnostic Model/ Dehydration Category	Sensitivity (95% CI)	Specificity (95% CI)	PPV (95% CI)	NPV (95% CI)	LR+ (95% CI)	LR− (95% CI)
**DHAKA Dehydration Score**						
Some dehydration	83% (80%, 87%)	52% (46%, 57%)	69% (65%, 73%)	71% (65%, 77%)	1.7 (1.5, 1.9)	0.33 (0.26, 0.41)
Severe dehydration	87% (80%, 94%)	57% (54%, 61%)	20% (16%, 24%)	97% (96%, 99%)	2.0 (1.8, 2.3)	0.23 (0.13, 0.40)
**DHAKA Dehydration Tree**						
Some dehydration	71% (67%, 75%)	72% (67%, 77%)	76% (72%, 81%)	66% (61%, 71%)	2.5 (2.1, 3.0)	0.40 (0.34, 0.47)
Severe dehydration	81% (73%, 89%)	67% (63%, 70%)	23% (18%, 28%)	97% (95%, 98%)	2.5 (2.1, 2.8)	0.28 (0.18, 0.44)

Abbreviations: CI, confidence interval; LR−, negative likelihood ratio; LR+, positive likelihood ratio; NPV, negative predictive value; PPV, positive predictive value.

### Recursive Partitioning to Derive the DHAKA Dehydration Tree

**FIGURE 3:**
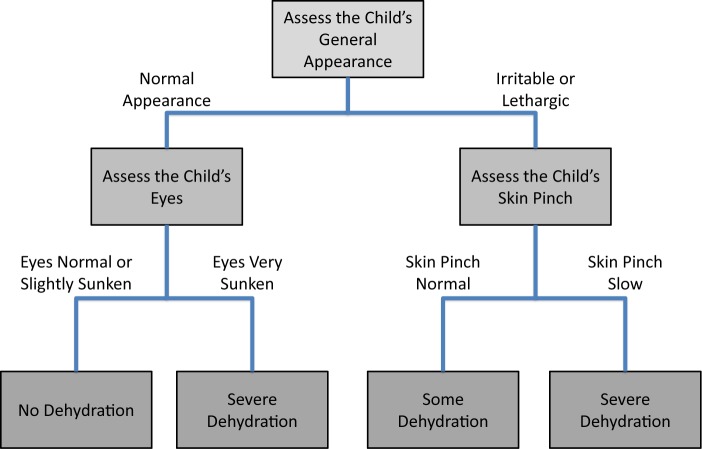
DHAKA Dehydration Decision Tree With Assigned Dehydration Categories

Recursive binary splitting followed by cross validation produced a final tree with 4 terminal nodes using just 3 variables: general appearance, eyes, and skin pinch (Figure 3). The AUC for this new DHAKA Dehydration Tree was 0.76 (95% CI = 0.71, 0.80) for severe dehydration and 0.75 (95% CI = 0.72, 0.78) for some (any) dehydration (Figure 2). For those children with a repeat clinical exam, the DHAKA Dehydration Tree had 90% agreement between independent raters, with a Cohen’s Kappa of 0.77 (95% CI = 0.67, 0.87). Table 4 demonstrates the proportion of children with no, some, or severe dehydration by DHAKA Dehydration Tree category, and Table 5 demonstrates the test characteristics of the DHAKA Dehydration Tree for assessing some and severe dehydration. For example, the DHAKA Dehydration Tree had a sensitivity of 81% and a specificity of 67%, a LR+ of 2.5, and a LR- of 0.28 for the outcome of severe dehydration.

The DHAKA Dehydration Tree, comprising 3 clinical signs of general appearance, eyes, and skin pinch, was also significantly accurate in diagnosing severe and any dehydration.

### Model Validation

The average AUC for the DHAKA Dehydration Score derived in the bootstrap samples was 0.80 for diagnosing severe dehydration, and the average performance of each of the bootstrap-derived models on the original dataset was 0.78, yielding an estimated optimism of 0.02 for the AUC. The average AUC for the DHAKA Dehydration Tree model derived in the bootstrap samples was 0.76 for the diagnosis of severe dehydration, and the average performance of each of the bootstrap-derived models on the original dataset was 0.74, yielding a similarly small estimated optimism of 0.02 for the AUC. The small optimism scores suggest that neither model is overly optimistic and both would likely perform similarly when tested in a new population of children.

Internal validation of the DHAKA Dehydration Score and Tree indicated the models would likely perform similarly well when tested in a new population of children.

## DISCUSSION

The DHAKA study has produced the first empirically derived and internally validated diagnostic model for assessing dehydration in children with acute diarrhea by general practice nurses in a resource-limited setting. The DHAKA Dehydration Score and DHAKA Dehydration Tree are clinical tools that may significantly assist nurses and other non-physician health workers to determine the best management strategy for children with acute diarrhea. Both the DHAKA Dehydration Score and DHAKA Dehydration Tree had significant positive and negative likelihood ratios, 90% inter-rater agreement, and modest optimism on bootstrap analysis.

Global health authorities recommend classifying children with acute diarrhea into 3 categories based on their initial clinical presentation, with significant differences in management based on the category assigned.^5^^-^^8^ Children classified as no dehydration (Plan A) receive only expectant outpatient management, with instructions given to continue breastfeeding as appropriate, provide the child with a normal diet, and encourage fluid intake. Children classified with some dehydration (Plan B) are rehydrated using ORS, an inexpensive but logistically intensive process. According to WHO guidelines, the child should be observed in the health facility for a minimum of 4 hours while the mother slowly spoons or drips 75 ml/kg of ORS into the child’s mouth, a few milliliters every minute.^10^ If the child still has some dehydration at the end of that period, the process is repeated for another 4 hours, requiring both a sufficient amount of space and an adequate number of health workers to observe this process over a prolonged time period. Finally, children with severe dehydration (Plan C) are resuscitated with IVF, which generally requires the child to be transferred to an inpatient facility. Not only is IVF more expensive and human resource-intensive than ORS (requiring careful vigilance to ensure the child is not overhydrated), but it also can cause more adverse events than ORS in children without severe dehydration, including seizures and death, and can lead to longer hospital lengths of stay.^4^

As such, the initial categorization of the dehydration status of a child with diarrhea has significant consequences, both to the individual child and to the health system as a whole. Inappropriate categorization, at best, will result in overutilization of precious health care resources. At worst, it will result in direct harm to the child. Despite the incredible importance of this initial diagnostic decision, however, the most accurate and reliable method for estimating the dehydration category of children with diarrhea in resource-limited settings has yet to be determined.

Accurate diagnosis of dehydration status in a child with diarrhea has significant consequences both to the individual child and to the health system as a whole.

Early dehydration scales were created based on expert opinion alone and never validated for their performance in children with diarrhea.^23^^,^^24^ In the past 2 decades, 4 clinical scales have been derived empirically using data from prospective cohorts of children against a valid criterion standard.^16^^,^^25^^-^^27^ All 4 scales, however, were developed in high- or middle-income countries based on the clinical assessments of highly skilled providers. It is unclear how well these scales might perform when used by less experienced providers in resource-limited settings, where the vast majority of diarrhea morbidity and mortality occurs. Clinical diagnostic models empirically derived in high- and middle-income countries may not perform as well in low-income countries where a higher proportion of acute diarrhea is caused by bacterial infections and where children tend to have higher rates of malnutrition. In addition, frontline providers in low-income countries are predominantly general practice nurses and health auxiliaries with limited training, which may also reduce the accuracy of clinical diagnostic models developed for use by physician specialists in high-resource settings. For the DHAKA study, all data were collected by research nurses without extensive experience in the management of dehydration in children, in order to ensure that the results would be as generalizable as possible to frontline health workers in other resource-limited settings.

Clinical diagnostic models derived in high- and middle-income countries may not perform as well in low-income countries.

Additionally, prior derivation studies of clinical diagnostic models have generally failed to provide explicit information about *how* study staff assessed each of the clinical signs of dehydration. For a clinical diagnostic model to perform as accurately in practice as it did in its derivation study, health workers worldwide must be able to assess each of the clinical variables within the model in the exact same way as the research staff who initially collected the study data. This means that it is not enough for a clinical diagnostic model to instruct health workers to assess for the presence or absence of sunken eyes or tears, but it must also specify how each of those signs was assessed by research staff in the original study. To achieve this objective, detailed protocols were developed *a priori* for the assessment of each of the clinical signs included in the DHAKA study, which have been included as a supplement to this article (Supplementary Appendix 2).

Finally, none of the 4 previously derived dehydration scales was based on cohorts of children large enough to develop a stable clinical diagnostic model. While a minimum number of 5–10 events per variable is required for the derivation of a stable model, the 4 prior studies each had 1–2 events per variable, making it unlikely for them to perform similarly in future datasets.^17^^,^^18^^,^^22^ Indeed, the Clinical Dehydration Scale, the only 1 of these 4 scales to be externally validated, has performed with mixed results in new populations of children.^11^^,^^12^^,^^27^^-^^31^ Not only is the DHAKA study the largest prospective study of dehydration assessment in children, enrolling more subjects than all 4 previously mentioned studies combined together, but it is also the first such study with more than 5 cases of severe dehydration per variable entered into the diagnostic model.

Worldwide, the most common clinical tool for assessing dehydration in children remains the WHO algorithm, which has been incorporated into the WHO IMCI guidelines and integrated into official ministry of health protocols in many low-income countries (Supplementary Appendix 1).^5^^,^^10^ While the WHO algorithm was originally created based on expert consensus, several recent studies have evaluated its capacity to discriminate between children with and without dehydration in both low- and high-income countries. A small study by Pringle et al. of 52 children presenting with acute diarrhea to 3 rural hospitals in East Africa found the WHO algorithm to be a poor predictor of severe dehydration in children, with an AUC of 0.58 (95% CI = 0.39, 0.78) for the prediction of moderate dehydration and 0.58 (95% CI = 0.41, 0.75) for the prediction of severe dehydration, neither of which were statistically different from chance.^11^ A somewhat larger study by Jauregui et al. of 113 patients presenting to an urban pediatric emergency department in the United States found the WHO algorithm to have an AUC of 0.61 (95% CI = 0.45, 0.77) for the prediction of moderate dehydration, also no different from chance.^13^ A final study conducted by Levine et al. among 178 children with acute diarrhea in Rwanda found the WHO algorithm to have a non-significant AUC of 0.65 (95% CI = 0.47, 0.83) for the prediction of severe dehydration when applied by general practice nurses.^12^ Overall, the study found the sensitivity of the WHO algorithm to be 67% and the specificity to be 68% for predicting severe dehydration in children.

Recent studies have found that the WHO algorithm does not discriminate well between children with and without dehydration.

As part of the DHAKA study, we have developed both a logistic regression model (referred to as the DHAKA Dehydration Score) and a recursive-partitioning model (referred to as the DHAKA Dehydration Tree) for the categorization of dehydration status in children with acute diarrhea. While the DHAKA Dehydration Score and DHAKA Dehydration Tree were found to have similar accuracy in our study, the latter may be easier to use by less experienced clinicians in resource-limited settings, since it does not require any computation and can be made into a completely visual decision tree. In addition, the DHAKA Dehydration Tree takes into account important interactions among variables. For instance, there appears to be an interaction between general appearance and sunken eyes, whereby very sunken eyes act as a strong predictor of severe dehydration in children with normal general appearance but add little to the diagnosis of severe dehydration in children with a lethargic appearance. Lack of the normal facial expressions seen in a happy, healthy child may make the eyes of a lethargic child appear quite sunken, even when they are not actually so.

The DHAKA Dehydration Tree may be easier to use than the Score by less experienced clinicians in resource-limited settings.

On the other hand, the DHAKA Dehydration Score can be more easily adapted to different settings, because it allows clinicians to choose their own cut-points for the 3 dehydration categories. For instance, if a clinician wanted a more sensitive test for severe dehydration, so as to be sure not to miss any children at risk for death, they could choose a lower cut-point than the score of 4, which we chose for this paper. Alternatively, if they wanted a more specific (though less sensitive) test, perhaps due to limitations in the availability of IVF, they could choose to use a higher cut-point for the diagnosis of severe dehydration.

The DHAKA Dehydration Score can be easily adapted to different settings.

While the DHAKA Dehydration Score and DHAKA Dehydration Tree share some elements with the current WHO algorithm, there are important differences. The primary difference is the absence of the clinical sign thirst, which requires differentiating between children who are refusing to drink because they are not thirsty and those who are refusing to drink because they are severely dehydrated. In addition, some providers may find either the DHAKA Dehydration Score or the DHAKA Dehydration Tree more intuitive and easier to use in practice than the WHO algorithm. Finally, both the DHAKA Dehydration Score and the DHAKA Dehydration Tree avoid the interaction between sunken eyes and lethargy, while the WHO algorithm does not.

### Strengths and Limitations

Our study population is not representative of all children in the world with diarrhea, most of whom never present to a health facility for clinical care, nor is it intended to be. However, we believe that our study population *is* reasonably representative of children with acute diarrhea who *do* present for medical care in low-income countries, and this is the population for which a clinical diagnostic model will be of most use. Since all clinical services are free, icddr,b cares for children from across the socioeconomic spectrum. Our study population includes a mix of children from both urban and rural settings, and a mix of children with both acute watery diarrhea and rice-water (typically cholera) diarrhea. Finally, about 90% of the children presenting to the rehydration unit at icddr,b arrive directly from home, with only 10% referred from other health facilities, making its case-mix more similar to a primary health center than a secondary referral hospital.

Moreover, since determination of the percent weight change with rehydration requires weighing children at regular intervals until they achieve a stable weight, it is not possible to conduct this study in a purely ambulatory setting, where patients are assessed only briefly and then discharged home or referred elsewhere. The rehydration unit at icddr,b provides the opportunity to observe ambulatory patients in a controlled setting long enough for the vast majority to achieve a stable weight before discharge home.

While the best physiologic criterion standard for dehydration remains the percent difference between pre-illness and admission weight, accurate pre-illness weights are rarely available for children in resource-limited settings. Instead, we used the percent weight change with rehydration as the criterion standard for percent dehydration in our study, which correlates almost perfectly with percent volume loss and has been used in nearly all prior studies of dehydration in children.^9^^,^^16^^,^^32^

We were able to assess the inter-rater reliability of the DHAKA Dehydration Score and DHAKA Dehydration Tree for only about half of patients enrolled in the study (those presenting during times of day when a second research nurse was available to perform a repeat exam). Even so, the lower bound of the 95% confidence intervals for the weighted kappa statistics for both the DHAKA Dehydration Score and DHAKA Dehydration Tree are greater than 0.60, which would generally be considered good reliability in the literature.

While internal validation using bootstrap sampling found good statistical reproducibility for both models, both the DHAKA Dehydration Score and the DHAKA Dehydration Tree require external validation in a new study population before they can be recommended for widespread clinical use. In addition, since these models were developed under relatively controlled conditions using data collected by dedicated research nurses with 4–6 years of clinical experience, they should be further assessed in other clinical settings with a variety of different providers in order to determine their generalizability.

## CONCLUSION

This is the first study to empirically derive stable clinical diagnostic models for dehydration in children with diarrhea. If validated in new cohorts of children, these new clinical tools should be incorporated into international and local guidelines for the management of childhood illness.

## Notes

**Peer Reviewed**
